# Stress in first-year medical students: a multidimensional analysis of emotional Intelligence, empathy, and cardiovascular health

**DOI:** 10.1186/s12909-025-08401-2

**Published:** 2025-12-12

**Authors:** Jaime A. Cespedes-Londono, Elizabeth Sanchez-Sanchez, Erika V. Ladino-Marin, Andrea C. Trompetero-Gonzalez, Alejandra Tordecilla-Sanders, Sara J. Guerrero-Leon, Catalina Arango-Holguin, Melissa Zambrano-Ferreira, Tatiana Camacho-Carvajal, Maria J. Rosero-Chamorro, Diana M. Ramos-Caballero, Kevin Maldonado-Cañón, Silvia Lopez-Guzman

**Affiliations:** 1 Pediatrics Research Group, Fundación Cardio Infantil-Instituto de Cardiología, Bogotá, 1113111 Colombia; 2 Clinical Investigation PhD candidate, School of Medicine and Health Sciences, Universidad del Rosario, Bogotá, 111221 Colombia; 3 Medical and Health Sciences Education Research Group, School of Medicine and Health Sciences, Universidad del Rosario, Bogota, 111221 Colombia; 4 Rehabilitation Science Research Group, Center for the Study of Physical Activity Measurement (CEMA), School of Medicine and Health Sciences, Universidad del Rosario, Bogota, 111221 Colombia; 5 School of Medicine and Health Sciences, Universidad del Rosario, Bogota, 111221 Colombia; 6 Unit on Computational Decision Neuroscience, Division of Intramural Research Program, National Institute of Mental Health, Bethesda, MD 20892 USA

**Keywords:** Medical students, Cardiovascular risk factors, Stress, Psychological, Empathy, Emotional intelligence

## Abstract

**Background:**

Medical students face elevated stress and mental health deterioration, driven by academic pressure, financial burden, and burnout. High stress also increases cardiovascular disease risk. In undergraduate systems like Colombia’s, students encounter these challenges earlier, yet few studies assess both psychological and physiological indicators in first-year cohorts. Limited context-specific data further hinders tailored support. This study explored links between perceived stress, emotional intelligence, empathy, and cardiovascular health among first-year medical students in Bogotá.

**Methods:**

We conducted a cross-sectional study in which participants completed validated surveys assessing perceived stress, emotional intelligence, and empathy. Cardiovascular health was evaluated using the American Heart Association’s Cardiovascular Health Index (CVHI) and waist-to-height ratio (WHtR). Data analysis included correlation matrices and regression models.

**Results:**

Among 195 medical students (62.1% female), 77.6% reported moderate-to-high stress. Although most participants had ideal CVHI scores, poor dietary and physical activity patterns were prevalent. Higher stress levels and less favorable cardiovascular indicators were observed alongside increased age, systolic blood pressure, and waist-to-height ratio. Emotional attention showed a positive association with stress and intermediate CVHI (PR = 5.34), and personal distress was associated with intermediate CVHI, particularly among students reporting elevated stress (PR = 2.52). Conversely, moderate physical activity, greater emotional clarity, and repair were associated with lower stress levels.

**Conclusions:**

Clarity, repair, and moderate physical activity appear protective against stress and cardiovascular risk in early medical training. In contrast, high emotional attention and personal distress increased vulnerability. Stress emerged as an important modifier of these relationships, underscoring its central role in student health. These findings support the implementation of locally tailored strategies to enhance emotional regulation and promote healthy behaviors in Colombian medical undergraduates.

**Supplementary Information:**

The online version contains supplementary material available at 10.1186/s12909-025-08401-2.

## Introduction

The transition into medical school is a particularly demanding period, marked by intense academic workloads, prolonged study hours, and emotional pressures [1, 2]. A growing body of evidence has documented high prevalence rates of stress, anxiety, and depression among medical students from the outset of their training, with consequences that extend beyond academic performance to reduced empathy, a critical capacity for sustaining student well-being and preserving the quality of future healthcare outcomes [3], as well as compromised physical and mental health, increased dropout risk, and potential long-term implications for professional practice [4, 5]. Studies suggest that at least half of medical students experience burnout [6] and alarmingly, indicate that burnout leads to between 3.5- and 6-times higher risk of self-harm and suicidal ideation [7, 8]. In parallel, unhealthy coping strategies—such as smoking, alcohol consumption, and physical inactivity—are frequently observed and are associated with elevated cardiovascular risk [9, 10] as well as other known cardiovascular risk factors such as dyslipidemia, elevated blood pressure, physical inactivity, and poor dietary habits [11, 12]. These challenges highlight the importance of psychoemotional health determinants such as empathy and emotional intelligence, known to buffer psychological distress, promote adaptive behaviors [13–15], and reduce cardiovascular risk [1, 16].

In response to the concerning health risks faced by medical students, many training institutions, particularly in high-income countries, developed early support systems focused on psychoemotional wellbeing and cardiovascular health promotion during medical education [17, 18] following a tailored approach based on descriptive local-specific evidence [1, 19, 20]. However, in low-and middle-income countries like Colombia, research on medical student health has primarily focused on cardiovascular risk factors alone [21, 22] rather than on psychoemotional health, often overlooking key distinctions across academic years [23]. This research gap has hindered our ability to detect when students are most vulnerable to cardiovascular and mental health deterioration, and limited the development of timely, evidence-based interventions.

In Colombia, first-year medical students often enter university directly after secondary school between the ages of 16 and 18, without prior higher education or preparatory college experience, which may affect their maturity, coping resources, and academic readiness. This developmental stage also coincides with a nationally elevated vulnerability to mental health disorders as identified in a recent mental health survey, showing that individuals aged 15–19 and 20–24 face a fivefold higher risk (IRR = 5.64 and 5.25, respectively) of anxiety [24]. Similarly, in the United States, emerging adults aged 18–25 report the highest prevalence of mental illness (33.7%), according to the 2024 Mental Health America report [25]. Longitudinal data showed that by 2021, over 60% of college students met criteria for at least one mental health condition, while their overall well-being declined significantly. These trends expose serious gaps in mental health support during the transition to adulthood [26]. While existing research emphasized the prevalence of academic stress and its physiological correlates [4, 5, 27], the simultaneous exploration of emotional coping and cardiovascular health appears to be less commonly addressed.

Understanding the dual vulnerability to psychoemotional distress and cardiovascular risk factors is of cardinal importance to further evidence-based comprehensive institutional support strategies. We aimed to assess the relationship between perceived stress, emotional intelligence, empathy and cardiovascular health in a cohort of first-year medical students enrolled at Universidad del Rosario – a private university in Bogota, Colombia. By integrating both psychological and physiological indicators, we provide a comprehensive overview of early vulnerabilities in medical training. Our study provides a baseline for future longitudinal follow-up, and to inform the design of targeted, evidence-based interventions that address the specific needs of younger medical students in medical programs worldwide.

## Methods

### Study design and participants

This cross-sectional study forms part of an ongoing longitudinal project titled *“Follow-up on Mind and Body Components in Medical Students at Universidad del Rosario.”* The project evaluates perceived stress, emotional intelligence, empathy, and cardiovascular health in first-year medical students, with follow-up planned after completion of basic medical training. Recruitment was conducted through in-class announcements during lectures, using voluntary response sampling across three enrollment waves between April 2019 and February 2020. Eligibility criteria required students to be enrolled in the first year of medical training, be 30 years of age or younger, have no pre-existing chronic medical conditions (e.g., hypertension, diabetes), and provide complete data on cardiovascular health, stress, and psycho-emotional measures. All participants provided written informed consent; students under the age of 18 additionally provided written assent along with consent from a parent or legal guardian.

### Procedures

Participants were assessed in person at the university’s campus infirmary. Trained medical students, physicians, and nurses performed physical measurements, including weight, height, waist circumference, blood glucose, total cholesterol (TC), and blood pressure. Participants also completed online self-reported surveys capturing sociodemographic data, emotional intelligence, empathy, perceived stress, and cardiovascular health variables. To ensure confidentiality, all sensitive information was collected directly through the Research Electronic Data Capture (REDCap) software [28, 29], anonymized it at entry, and restricted access to the research team. Senior medical students, supervised by researchers, assisted with physical measurements. None of the research team held teaching or evaluative roles over participants, reducing the risk of social desirability bias or fear of judgment.

Participants self-reported their sex. Gender identity was not collected. Given that some outcomes may reflect both biological and behavioral influences, the term *sex* is used throughout the analysis without distinguishing it from gender. This choice reflects the structure of the available data while acknowledging the conceptual distinction between sex and gender.

### Variables

#### Emotional intelligence, empathy, and perceived stress

Emotional intelligence was assessed using the Trait Meta-Mood Scale-24 (TMMS24) [30], validated in Colombia [31]. The instrument includes 24 items across three dimensions: Attention, Clarity, and Repair, rated on a 5-point Likert scale [1–5]. Subscale scores were obtained by adding items. Following the original scoring recommendations, each domain was categorized as low, adequate, or high using sex-specific cut-off points [32]. Empathy was assessed using the Interpersonal Reactivity Index (IRI) [33], validated in Colombia [34]. The 28-item tool includes four subscales: Fantasy, Perspective Taking, Empathic Concern, and Personal Distress. Items are rated on a 5-point Likert scale (0–4), with subscale scores ranging from 0 to 28. Perceived stress was measured using the Perceived Stress Scale (PSS-14) [35] validated for medical students in Colombia [36]. Scores range from 0 to 56 points, categorized as: Low perceived stress: <18, Moderate perceived stress: 19–37, and High perceived stress: ≥38 [37].

#### Cardiovascular health

##### Cardiovascular health index (CVHI)

The CVHI includes four health behaviors (Body Mass Index (BMI), smoking, diet, physical activity) and three biological factors (blood glucose, TC, blood pressure), each scored as poor (0), intermediate (1), or ideal (2), for a total score of 0–14. Categories follow AHA guidelines: poor (0–4), intermediate (5–9), and ideal (10–14) [38].

BMI was calculated as weight (kg)/height² (m²), following ISAK standards, using a SECA 876^®^ scale and a SECA 264^®^ stadiometer. Smoking status was assessed via self-report and categorized as ideal (never or quit > 1 year), intermediate (quit < 1 year), or poor (current smoker). Diet was assessed using a 10-item subset of a validated 26-item Spanish Food Frequency Questionnaire [39]. To ensure contextual relevance, we adapted the CVHI diet criteria to three components: fruits and vegetables, whole grains, and sugar-sweetened beverages. For interpretability purposes, intake was categorized by daily frequency: ideal thresholds were ≥ 5 times/day, ≥ 1 time/day, and ≤ 1 time/day, respectively. Each ideal component scored 1 point (0–3 total), with the overall diet classified as poor (0), intermediate (1-2), or ideal (3). Physical activity was measured using the short-form International Physical Activity Questionnaire (IPAQ) [40], which records weekly frequency and duration of walking, moderate, and vigorous activity.

Blood glucose and TC levels were assessed from 40 µL capillary blood samples after 8–12 h fasting. Glucose was measured with an Accu-Chek^®^ Inform II (CV 1.8%), and cholesterol with an Accutrend^®^ Plus (CV 1.1–3.8%). Lipid status was evaluated using TC, in line with the AHA’s CVHI definition [38]. LDL cholesterol was not collected. Blood Pressure (BP) was measured twice using a Riester Ri-Champion^®^ N sphygmomanometer (± 3 mmHg accuracy) after a 5-minute seated rest, with a 20-minute interval between readings. The average was recorded.

##### Waist-to-Height ratio (WHtR)

WHtR was calculated as waist circumference (cm)/height (cm). Colombian WHtR risk thresholds were defined using sex- and age-specific cut-off values reported in the literature [41, 42]. Because no reference values existed for individuals aged 18–19.9 years, we interpolated linearly between the adjacent categories (17–17.9 and 20–24.9 years). We used the midpoint of the surrounding thresholds, yielding cut-offs of 0.4525 for males and 0.470 for females. Values above these thresholds were classified as high CVD risk.

### Statistical analysis

Continuous variables were assessed for normality using histograms and the Kolmogorov–Smirnov test and presented as means with standard deviations or medians with interquartile ranges (IQR). Discrete variables were reported as absolute and relative frequencies. Data visualization included bar plots, box plots, and correlation plots. Differences across sexes were analyzed using the Wilcoxon rank sum test (nonparametric data), ANOVA (parametric data), and Pearson’s Chi-squared test (categorical variables). Spearman’s rank correlation assessed relationships between continuous variables [43]. Univariable and multivariable Robust Poisson regression for frequent outcomes using standard errors estimated Prevalence Ratios (PR) for intermediate CVHI, low emotional clarity, low emotional repair, and adequate emotional attention [44], while logistic regression modeled high PSS and high cardiovascular risk based on WHtR, and lineal regression modeled PSS as a continuous variable. Overdispersion adjustments and multicollinearity checks (variance inflation factors (VIFs)) ensured robustness. Model performance was evaluated using the c-statistic, Hosmer-Lemeshow, and deviance goodness-of-fit tests. Standardization and transformations were applied to improve model interpretability.

We systematically evaluated the risk of overfitting in our regression models using the events-per-variable (EPV) criterion and Poisson model-specific diagnostic approaches such as the ratio of the Pearson χ² statistic to the degrees of freedom [45] and the Applied Econometrics with R (AER) dispersion test, which tests the null hypothesis of equidispersion in Poisson generalized linear models against the alternative of overdispersion or underdispersion [46]. Given the small number of outcome events for high PSS, we used Firth’s penalized logistic regression model, which reduces small-sample bias and prevents issues like separation that can occur in sparse data [47]. All analyses, conducted in R Studio (RRID: SCR_001905) [48]. We report 95% confidence intervals and define statistical significance at the level of *p* < 0.05.

## Results

### Study population

A total of 393 first-year medical students were invited to participate during the study period, of whom 304 accepted. After eligibility screening, 195 participants were included, representing 49.9% of the admitted cohort students into the first year (See Appendix Fig. 1). As shown on Table 1 and 62.1% of our sample were female with a median age of 18.0 years (Q1: 17.0, Q3: 19.0) and no significant age difference between sexes.

**Table 1:** Demographics, emotional intelligence, empathy, perceived stress, and cardiovascular health

	Female (*N* = 121)	Male (*N* = 74)	Total (*N* = 195)	*p* value
Demographic characteristics				
Age, median (Q1, Q3)	18.0 (17.0, 19.0)	18.0 (17.0, 18.0)	18.0 (17.0, 19.0)	0.370
Emotional intelligence				
*Attention (score)*	25.0 (6.9)	24.2 (7.7)	24.7 (7.2)	0.537
Attention Classification				0.030
Little	46 (46.9%)	25 (44.6%)	71 (46.1%)	
Too much	6 (6.1%)	11 (19.6%)	17 (11.0%)	
Adequate	46 (46.9%)	20 (35.7%)	66 (42.9%)	
*Clarity (score)*	23.0 (6.2)	25.0 (5.9)	23.7 (6.2)	0.059
Clarity Classification				0.636
Low	60 (61.2%)	31 (55.4%)	91 (59.1%)	
Adequate	35 (35.7%)	24 (42.9%)	59 (38.3%)	
Excellent	3 (3.1%)	1 (1.8%)	4 (2.6%)	
*Repair (score)*	25.0 (5.8)	27.0 (6.6)	25.7 (6.2)	0.046
Repair Classification				0.058
Low	46 (46.9%)	16 (28.6%)	62 (40.3%)	
Adequate	46 (46.9%)	33 (58.9%)	79 (51.3%)	
Excellent	6 (6.1%)	7 (12.5%)	13 (8.4%)	
Empathy				
*Perspective Taking Score*	13.6 (3.6)	14.3 (4.0)	13.9 (3.8)	0.319
*Fantasy Scale Score*	11.6 (4.5)	11.5 (5.2)	11.6 (4.7)	0.890
*Empathic Concern Score*	10.7 (3.2)	10.3 (3.9)	10.6 (3.5)	0.553
*Personal Distress Score*	9.1 (3.0)	9.4 (3.7)	9.2 (3.3)	0.535
Perceived stress				
Perceived Stress Scale Total	27.2 (8.1)	23.2 (8.9)	25.6 (8.6)	0.005
PSS Classification				0.158
High	9 (9.7%)	5 (8.5%)	14 (9.2%)	
Moderate	68 (73.1%)	36 (61.0%)	104 (68.4%)	
Low	16 (17.2%)	18 (30.5%)	34 (22.4%)	
Cardiovascular health				
Total CVHI, median (Q1, Q3)	11.0 (10.0, 12.0)	11.0 (10.0, 12.0)	11.0 (10.0, 12.0)	0.575
CVHI Classification				0.484
Ideal	89 (77.4%)	58 (81.7%)	147 (79.0%)	
Intermediate	26 (22.6%)	13 (18.3%)	39 (21.0%)	
Waist-to-Height Ratio	0.5 (0.0)	0.4 (0.1)	0.5 (0.1)	0.877
Waist-to-Height Risk Category				0.820
High Risk	49 (43.4%)	30 (41.7%)	79 (42.7%)	
Low Risk	64 (56.6%)	42 (58.3%)	106 (57.3%)	

### Emotional intelligence, empathy, and perceived stress

Regarding the emotional intelligence dimensions, participants reported an average of 24.7 (SD: 7.2) for attention to feelings, 23.7 (SD: 6.2) for clarity of feelings, and 25.7 (SD: 6.2) for mood repair. Most were classified as having little or adequate attention, with males significantly reporting higher levels of attention (19.6%) compared to females (6.1%) (*p* = 0.03). Most of both female (61.2%) and male (55.4%) were classified as having low levels of emotional clarity, with no significant sex differences (*p* = 0.636). In terms of mood repair, although differences were not statistically significant (*p* = 0.058), males showed a higher proportion of excellent (12.5%), and adequate (58.9%) repair compared to females (6.1% and 46.9%, respectively). Turning to empathy, the average scores for perspective taking, fantasy, empathic concern, and personal distress were 13.9 (SD: 3.8), 11.6 (SD: 4.7), 10.6 (SD: 3.5), and 9.2 (SD: 3.3), respectively. There were no statistically significant differences between sexes for any domain (Table 1).

Considering perceived stress (PSS), the average was 25.6 (SD: 8.6) with females reporting significantly higher scores than males (27.2 vs. 23.2; *p* = 0.005). Most participants were classified as experiencing moderate stress (68.4%), followed by low (22.4%) and high (9.2%) stress. Although the differences between sexes were not statistically significant (*p* = 0.158), low stress levels were more frequently reported by males (30.5%) compared to females (17.2%), whereas females more often fell into the moderate category (73.1% vs. 61.0%) (Table 1).

### Cardiovascular health

**Fig. 1:**
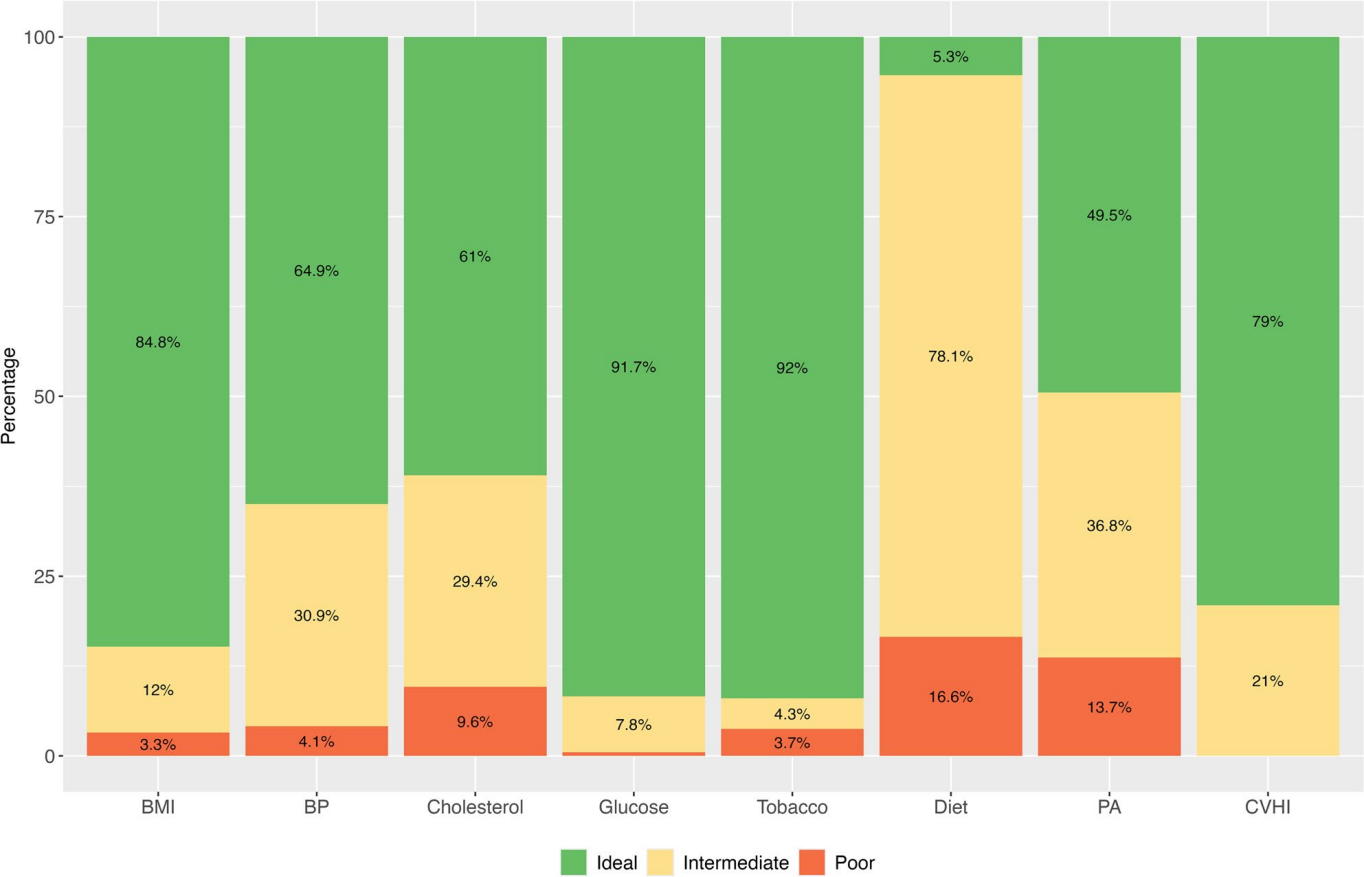
Cardiovascular Health Index (CVHI) components. Note: PA: Physical Activity, BP: Blood Pressure, BMI: Body mass index, CVHI: Cardiovascular Health Index. Component scores: 0 (poor), 1 (intermediate), or 2 (ideal), maximum total score: 14. CVHI categories: poor (0–4 points), intermediate (5–9 points), ideal (10–14 points)

The median CVHI score was 11.0 (IQR: 10.0–12.0) for both sexes, with 79.0% of participants classified as having an ideal cardiovascular health index (CVHI) and 21.0% as intermediate, showing no significant sex differences (*p* = 0.484). Significant sex differences were observed in the blood pressure, cholesterol, and physical activity components of the CVHI. A notably higher proportion of females met ideal criteria for blood pressure (80.2% vs. 39.7%, *p* < 0.001), whereas males more frequently achieved ideal cholesterol levels (70.8% vs. 54.8%, *p* = 0.019) and ideal physical activity (65.8% vs. 39.3%, *p* = 0.002) (Fig. 1). No significant differences were found in BMI, glucose, tobacco use, or diet components. Additionally, compared to males, females had lower average systolic (110.9 vs. 123.0 mmHg, *p* < 0.001) and diastolic blood pressure (69.5 vs. 74.0 mmHg, *p* < 0.001), lower glucose levels (87.9 vs. 91.7 mg/dL, *p* = 0.003), and higher minimum heart rate (78.4 vs. 73.3 bpm, *p* = 0.008). Cholesterol levels were slightly higher in females (164.0 vs. 157.5 mg/dL, *p* = 0.039). No significant differences were observed in BMI, tobacco use, or dietary intake indicators (grains, beverages, or fruits). A detailed description of the CVHI components and physiological variables can be found in Table S1.

Average waist circumference was significantly higher in males (78.2 cm) than in females (72.1 cm; *p* < 0.001). The mean WHtR did not differ between females and males (0.50 vs. 0.49; *p* = 0.877). Overall, 42.7% of participants were classified as having high WHtR CVD risk, with no significant difference between sexes (Table 1).

Regarding physical activity, participants reported a median of 60 min per week of moderate physical activity and 60 min of vigorous activity, with interquartile ranges indicating high variability. Walking was the most frequently reported activity, with a median of 280 min per week and a total median energy expenditure of 1927.5 MET-min/week across all activity domains. Despite this, 38.8% of participants reported prolonged sitting times (≥ 481 min per day), and the overall median sitting time was 480 min per day. Males reported significantly higher levels of moderate physical activity compared to females, both in minutes per week (median: 120.0 vs. 39.8, *p* = 0.006) and corresponding METs (480.0 vs. 159.2, *p* = 0.006). Additionally, males had a higher total physical activity level (median METs: 2488.0 vs. 1737.0, *p* = 0.032). No significant sex differences were observed in sitting time or walking activity (Table S2).

### Relationship between cardiovascular health index and other health factors

**Fig. 2:**
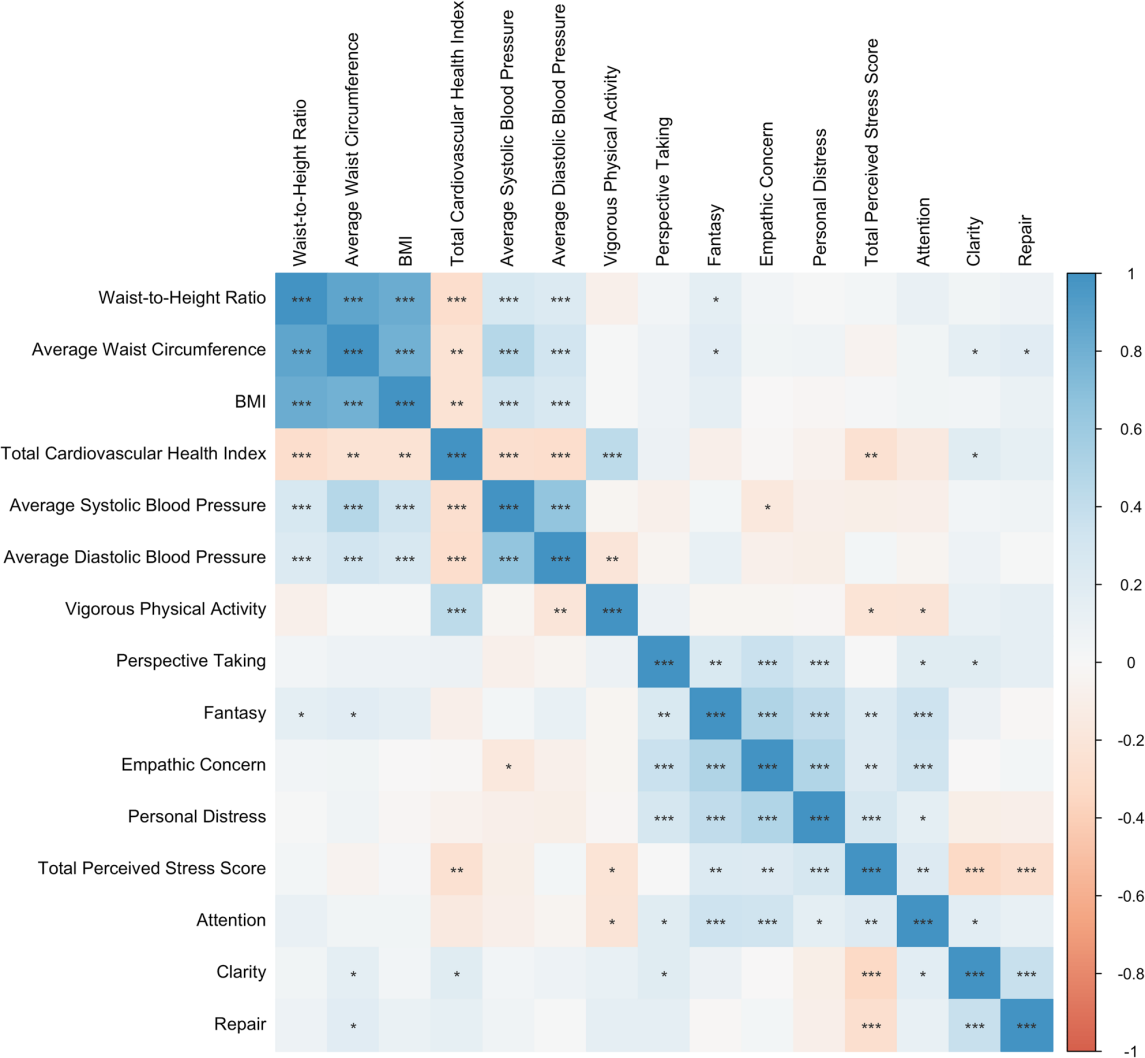
Correlation plot. Note: *p*-value ***<0.001; **0.01, *0.05. IRI subscales: Fantasy, Perspective, Empathic Concern, Personal Distress, TMMS-24 dimensions: Attention, Clarity and Repair

While in this generally healthy population CVHI was high, we nonetheless found that CVHI was negatively correlated with known cardiovacular risk factors such as adiposity indicators—BMI (*r* = − 0.239), waist circumference (*r* = − 0.231), and WHtR (*r* = − 0.280)—as well as with systolic (*r* = − 0.273) and diastolic blood pressure (*r* = − 0.281), and perceived stress (*r* = − 0.256). In contrast, CVHI showed positive correlations with vigorous physical activity (*r* = 0.438). Interestingly, we found that CVHI was marginally correlated with emotional clarity (*r* = 0.192), a specific dimension of emotional intelligence (Fig. 2). We found no correlation between CVHI and emotional attention or mood repair, the other two dimensions of emotional intelligence we measured.

### Factors associated with poor cardiovascular and psychosocial health outcomes

#### Emotional intelligence

Low emotional clarity was significantly associated with more sedentary time (PR: 0.76, 95% CI: 0.63–0.91, *p* = 0.003), lower fantasy scores (PR: 0.48, 95% CI: 0.35–0.66, *p* < 0.001), higher personal distress (PR: 1.73, 95% CI: 1.13–2.66, *p* = 0.012), and increased perceived stress (PR: 1.19, 95% CI: 1.02–1.39, *p* = 0.024). Low mood repair was associated with lower waist-to-height ratio (PR: 0.72, *p* = 0.015), higher heart rate (PR: 1.20, *p* = 0.046), lower moderate physical activity (PR: 0.71, *p* = 0.024), greater sitting time (PR: 0.80, *p* = 0.027), and higher perceived stress (PR: 3.93, 95% CI: 1.54–10.04, *p* = 0.004).Adequate attention to feelings was positively associated with walking time (PR: 1.20, 95% CI: 1.05–1.37, *p* = 0.006), fantasy scores (PR: 1.82, 95% CI: 1.05–3.15, *p* = 0.032), and emotional clarity (PR: 2.50, 95% CI: 1.01–6.17, *p* = 0.047), but inversely related to vigorous physical activity (PR: 0.54, *p* = 0.007) (See Table 2).

**Table 2:** Multivariable robust Poisson regression models

Multivariable robust Poisson regression models	PR	95% CI	Robust SE	*P*-value
Outcome: Intermediate CVHI EPV = 4.9; Pearson χ²/df = 0.78; AER dispersion test: 0.83, *p* = 0.998				
Waist-to-Height Ratio (+ 1 SD)	1.38	1.07–1.79	0.131	0.013
Minimum Heart Rate (+ 1 SD)	1.63	1.11–2.4	0.198	0.014
Minutes Sitting Per Day (+ 1 SD)	1.09	0.77–1.55	0.177	0.620
Perspective Taking Score (Log)	2.01	0.59–6.8	0.622	0.262
Personal Distress Score (Log)	0.98	0.33–2.91	0.557	0.967
Total Perceived Stress Score (+ 1 SD)	0.09	0.01–0.67	1.042	0.019
Emotional Attention (Log)	5.34	1.26–22.61	0.737	0.023
Emotional Clarity (+ 1 SD)	0.67	0.45–1.45	0.205	0.048
PD Score (Log): Total PSS (+ 1 SD)	2.52	1.11–5.73	0.419	0.027
Outcome: Low clarity EPV = 11.4; Pearson χ²/df = 0.44; AER dispersion test: 0.43, *p* = 1				
METS of Vigorous Physical Activity (+ 1 SD)	0.95	0.78–1.16	0.102	0.609
METS of Moderate Physical Activity (+ 1 SD)	0.96	0.79–1.17	0.100	0.715
METS of Walking (+ 1 SD)	0.92	0.77–1.09	0.089	0.341
Waist-to-Height Ratio (+ 1 SD)	0.93	0.76–1.15	0.105	0.511
Minutes Sitting Per Day (+ 1 SD)	0.76	0.63–0.91	0.094	0.003
Fantasy Score (Log)	0.48	0.35–0.66	0.165	< 0.001
Personal Distress (Log)	1.73	1.13–2.66	0.220	0.012
Total Perceived Stress Score (+ 1 SD)	1.19	1.02–1.39	0.078	0.024
Outcome: Low repair EPV = 8.4; Pearson χ²/df = 0.65; AER dispersion test: 0.64, *p* = 1				
Waist-to-Height Ratio (+ 1 SD)	0.72	0.55–0.94	0.134	0.015
Minimum Heart Rate (+ 1 SD)	1.20	1–1.44.44	0.092	0.046
Vigorous Physical Activity min/day (+ 1 SD)	1.00	0.7–1.42	0.180	0.994
Moderate Physical Activity min/day (+ 1 SD)	0.71	0.53–0.96	0.153	0.024
Walking min/day (+ 1 SD)	1.11	0.92–1.35	0.098	0.267
Minutes Sitting Per Day (+ 1 SD)	0.80	0.65–0.97	0.102	0.027
Total Perceived Stress Score (Log)	3.93	1.54–10.04	0.479	0.004
Outcome: Adequate attention EPV = 9.4; Pearson χ²/df = 0.53; AER dispersion test: 0.54, *p* = 1				
Waist-to-Height Ratio (+ 1 SD)	1.04	0.84–1.28	0.107	0.741
Vigorous Physical Activity min/day (+ 1 SD)	0.54	0.35–0.85	0.226	0.007
Moderate Physical Activity min/day (+ 1 SD)	0.98	0.77–1.25	0.125	0.872
Walking min/day (+ 1 SD)	1.20	1.05–1.37	0.067	0.006
Minutes Sitting Per Day (+ 1 SD)	0.82	0.66–1.03	0.116	0.095
Fantasy Score (Log)	1.82	1.05–3.15	0.280	0.032
Clarity (Log)	2.50	1.01–6.17	0.462	0.047

*AER* Applied Econometrics with R, *EPV *events-per-variable, *Pearson χ²/df* ratio of the Pearson χ² statistic to the degrees of freedom

#### Stress

**Fig. 3:**
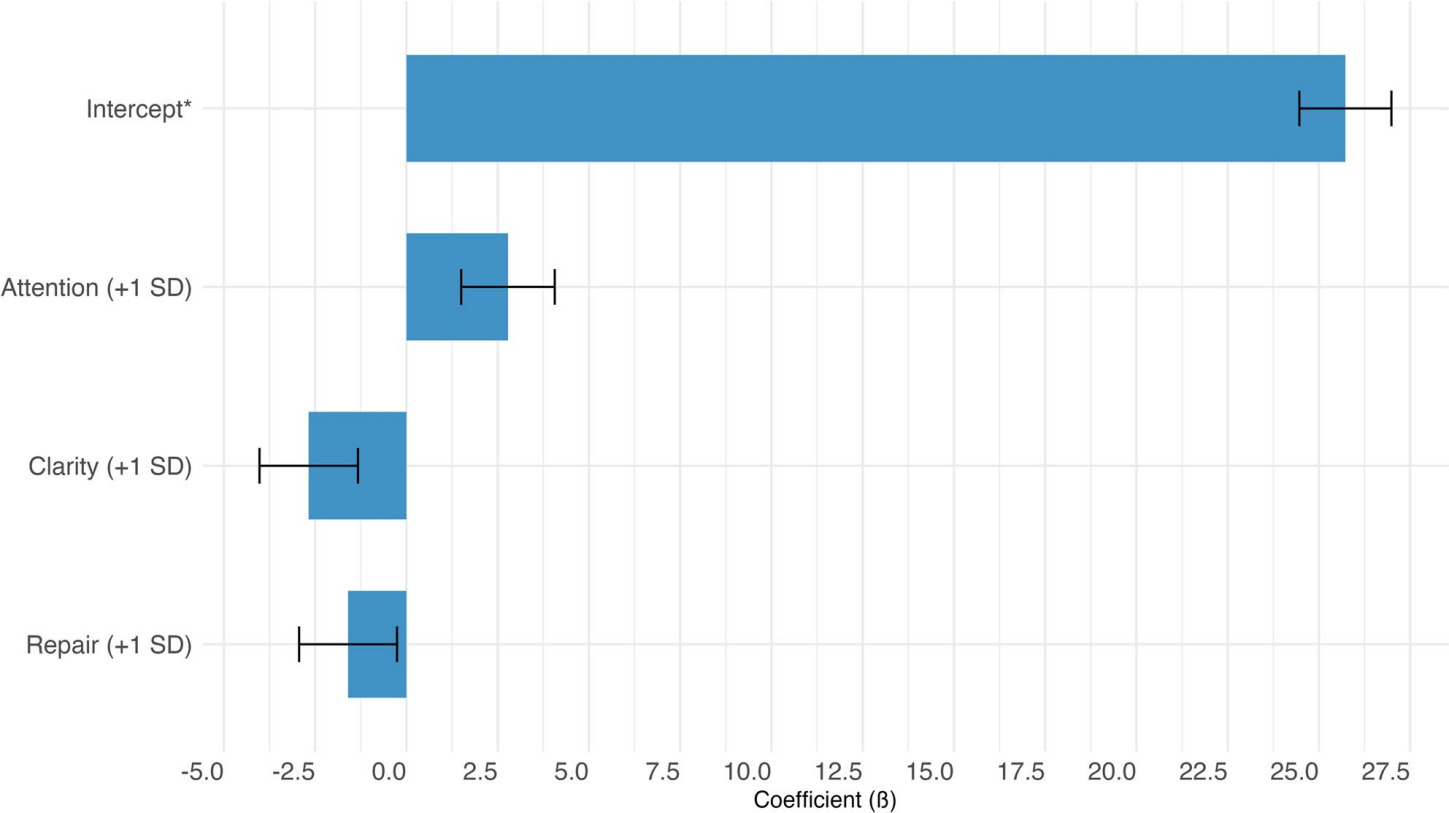
Multivariable linear regression – Outcome: Perceived Stress Score (PSS)

In multivariable logistic regression, high perceived stress was associated with older age (PR: 2.07, 95% CI: 1.10–4.16, *p* = 0.028), higher waist-to-height ratio (PR: 1.93, 95% CI: 1.02–3.70, *p* = 0.038), and lower moderate physical activity (PR: 0.17, 95% CI: 0.03–0.59, *p* = 0.022) (Tables 3). On the other hand, multivariable linear regression showed that greater emotional clarity (β = −2.68, *p* < 0.001) and better mood repair (β = −1.60, *p* = 0.02) were significantly associated with lower perceived stress scores (PSS). In contrast, higher attention to feelings was linked to increased stress levels (β = 2.78, *p* < 0.001) (Fig. 3).

**Table 3:** Multivariable logistic regression models

Multivariable logistic regression models	PR	95% CI	-	*P*-value
Outcome: High PSS* EPV = 4.7				
Age (+ 1 SD)	1.93	1.07, 3.70	-	0.029
Waist-to-Height Ratio (+ 1 SD)	1.85	1.02, 3.35	-	0.042
MPA min_day (+ 1 SD)	0.23	0.04, 0.71	-	0.006
Outcome: High cardiovascular risk based on WHtR EPV = 11.3				
Male	0.46	0.10, 1.85	-	0.28
Body Mass Index	2.86	1.95, 4.76		< 0.001
Age (+ 1 SD)	0.50	0.25, 0.95	-	0.037
Average Systolic Pressure (+ 1 SD)	1.63	0.75, 3.66	-	0.22
Sitting min_day (+ 1 SD)	0.93	0.53, 1.62	-	0.79
MPA min_day (+ 1 SD)	0.47	0.22, 0.89		0.033
Emotional Attention (+ 1 SD)	1.34	0.75, 2.44	-	0.33

*Firth’s penalized logistic regression

As we indicated previously, only 21% of our participants had less than ideal CVHI. To identify factors related to intermediate CVHI, we ran a robust Poisson regression model. We found that intermediate CVHI was significantly associated with higher waist-to-height ratio (PR: 1.38, 95% CI: 1.07–1.79, *p* = 0.013), elevated minimum heart rate (PR: 1.63, 95% CI: 1.11–2.40, *p* = 0.014), but also with higher attention to feeling scores (PR: 5.34, 95% CI: 1.26–22.61, *p* = 0.023). Conversely, higher total perceived stress score (PR: 0.09, 95% CI: 0.01–0.67, *p* = 0.019) and emotional clarity (PR: 0.67, 95% CI: 0.45–1.00, *p* = 0.048) were inversely associated with this outcome. A significant interaction term (Personal Distress × PSS) also showed increased probability of intermediate CVHI (PR: 2.52, 95% CI: 1.11–5.73, *p* = 0.027).

A high cardiovascular risk based on WHtR was less frequent among older individuals (PR: 0.50, *p* = 0.037 per one SD increase) and those with more minutes of MPA per day (PR: 0.47, *p* = 0.033 per one SD increase). Conversely, it was more frequent among individuals with a higher BMI (PR: 2.86, *p* < 0.001 per one unit increase).

## Discussion

This cross-sectional study explored associations between perceived stress, emotional intelligence, empathy and cardiovascular health among medical students. Elevated stress levels were observed, particularly in female students and, were associated with sedentary behavior and traditional cardiovascular risk factors. Emotional clarity, emotional repair, and physical activity were associated with lower stress, while heightened emotional attention was linked to increased stress levels.

Most of our participants reported moderate to high stress levels, consistent with findings from Brazil, Saudi Arabia, Peru, and the U.S [49–52]. Elevated stress in this population has been associated with academic overload, competition, and inadequate coping mechanisms [53, 54], and may be exacerbated by insufficient institutional support [55, 56], which has been linked to higher prevalence of psychological and physical conditions such as anxiety, insomnia, and hypertension [57]. At the same time, personal and contextual strategies—such as physical activity and emotional intelligence—are associated with lower stress levels among medical students [58–60]. Accordingly, our study demonstrated that moderate physical activity was significantly associated with reduced perceived stress. Furthermore, clarity and repair are associated negatively with stress consistent with findings by Barraza-López [61], supporting their potential role as correlates of lower stress [55, 56].

In contrast, some factors, notably cardiovascular health indicators, showed positive correlations with stress. Although many students had adequate cardiovascular parameters, higher stress was linked to poor diet and sedentary behavior, consistent with broader college populations [62]. Regarding emotional intelligence, attention was related to higher perceived stress, consistent with evidence that excessive attention, particularly without clarity and repair, is linked to poorer adjustment, distress, and rumination, whereas clarity and repair foster resilience and mitigate stress [63–66]. Empathic concern and personal distress were respectively associated with blood pressure and intermediate CVHI prevalence, indicating that psychosocial factors may be linked to both mental and physical health— an area that has received limited attention but is relevant to medical education [67, 68]. According to the literature, other risk factors for higher stress included early academic stage, pre-existing illness, low social support, dissatisfaction, and substance use [69–71]. Altogether, these results suggest associations that may inform efforts to promote emotional regulation skills and healthy behaviors in medical students, while also guiding future research.

Cardiovascular risk factors among medical students have been documented globally. Similar to U.S. findings reporting elevated cholesterol and blood pressure in medical students [72], our study found poor dietary habits, low physical activity, and signs of cardiometabolic risk in a young sample (17–19 years old, mostly female), Although most students achieved ideal CVHI scores, nearly 43% met WHtR criteria for elevated cardiovascular risk, reflecting a considerable burden of central adiposity within this cohort. In addition, studies conducted in Italy, Brazil, Peru, and the UAE revealed low rates of ideal CVHI, high physical inactivity, elevated cholesterol, and obesity, particularly among female students [73–76]. These variations may be influenced by age, assessment methods, and economic status, with sharper declines observed among low socioeconomic Brazilian youth and worsening indicators among Italian medical students [77]. In Colombia, the institutional context—where medical students can enter directly from high school—may also impact emotional maturity and the accuracy of self-reported data.

As an explanatory mechanism in our context, participants enter a high-pressure environment at a young age, while their prefrontal cortex is still maturing [78], which could limit emotional maturity and coping strategies [15, 63]. The transition from a structured high school setting to the demanding, high-stakes culture of medical education may represent an abrupt academic and social shift, potentially increasing perceived stress and emotional dysregulation [79–81]. Students with high emotional sensitivity or empathy may be especially vulnerable, as their attunement to others can intensify the impact of academic, social, and interpersonal challenges [15, 63, 82]. As students’ progress, the cumulative effects of academic demands, internalized performance pressure, and limited institutional support contribute to deteriorating mental health and less adaptive coping [80, 83]. Despite this, many students maintain external academic success while their psychological well-being declines [81, 83]. Emotional strain is often minimized or stigmatized within the medical culture, making it harder for vulnerable students to access help and further reinforcing patterns of silence [81, 82]. In this context, evidence-based non-pharmacological strategies such as mindfulness-based stress reduction, resilience training programs, self-care mind–body groups, and brief heart rate variability coherence practices like the HeartMath ‘Freeze-Frame’ technique have shown efficacy in reducing stress and enhancing wellbeing in medical students and related populations [82–88].

### Strengths and limitations

This study has several strengths. First, it included a relatively large sample size of first-year medical students, representing nearly half of the admitted cohort during the study period. Second, the study forms part of an ongoing longitudinal project, which will allow for future follow-up analyses to better understand temporal changes and causal relationships between stress, emotional intelligence, empathy, and cardiovascular health. Finally, the use of standardized, validated instruments and objective physiological measurements enhances the reliability and reproducibility of our findings.

However, some limitations must be acknowledged. Academic performance, which may correlate with stress and psycho-emotional variables, was not assessed and could provide important additional context. Similarly, gender identity was not evaluated; our analysis relied on self-reported sex, limiting the inclusivity and scope of our findings considering contemporary approaches to gender in health research. In addition, the cross-sectional design precludes causal inference, and the reliance on self-reported measures for psycho-emotional variables may introduce reporting bias. In terms of cardiovascular risk assessment, we followed the CVHI metrics, using total cholesterol as the lipid component; however, LDL-C was not available. Recent evidence suggests LDL-C may provide more accurate risk stratification, and newer studies question a strictly linear causal link between cholesterol levels and cardiovascular outcomes. This underscores the need for cautious interpretation of our lipid findings [89–91]. Despite these limitations, the study provides valuable evidence to guide interventions targeting the psycho-emotional and cardiovascular health of medical students during the critical transition into medical training.

## Conclusions

This study ratifies the high prevalence of stress among medical students. MPA and emotional regulation abilities - clarity and repair - were associated with reduced stress levels. However, many students—particularly those who enter medical training at a younger age—face significant challenges characterized by unhealthy behaviors and psychosocial vulnerabilities. Despite adequate CVHI scores, early indicators of poor lifestyle habits and emotional dysregulation were evident. These findings emphasize the urgent need to integrate individual-level support with structural reforms in medical education that foster emotional intelligence traits and address the cultural stigmatization of mental health.

While the study provides meaningful insights, its cross-sectional design limits causal conclusions, in addition, self-reported measures of emotional well-being may be biased, especially given the young age and limited life experience of Colombian medical students. Unmeasured variables—such as genetics, sleep quality, or social support—could also influence the results, and the findings may not generalize to other populations. Future research should use longitudinal designs and randomized controlled trials to explore causal pathways between emotional intelligence, stress, and cardiovascular health. Including biomarkers like cortisol and expanding the sample to other healthcare populations could strengthen and broaden the applicability of these findings.

## Supplementary Information

Supplementary Material 1.

Supplementary Material 2.

## Data Availability

The dataset supporting this article’s conclusions is available in the Figshare repository: [10.6084/m9.figshare.28254791] 10.6084/m9.figshare.2825479
